# Repurposing FIASMAs against Acid Sphingomyelinase for COVID-19: A Computational Molecular Docking and Dynamic Simulation Approach

**DOI:** 10.3390/molecules28072989

**Published:** 2023-03-27

**Authors:** Aliza Naz, Sumbul Asif, Khairiah Mubarak Alwutayd, Sara Sarfaraz, Sumra Wajid Abbasi, Asim Abbasi, Abdulkareem M. Alenazi, Mohamed E. Hasan

**Affiliations:** 1 National Center for Bioinformatics, Quaid-i-Azam University, Islamabad 45320, Pakistan; 2 Department of Bioinformatics and Biotechnology, International Islamic University, Islamabad 44000, Pakistan; 3 School of Interdisciplinary Engineering and Sciences, National University of Sciences and Technology, Islamabad 44000, Pakistan; 4 Department of Biology, College of Science, Princess Nourah bint Abdulrahman University, P.O. Box 84428, Riyadh 11671, Saudi Arabia; 5 Department of Bioinformatics, Kohsar University Murree, Murree 47150, Pakistan; 6 Department of Biological Sciences, National University of Medical Sciences, Rawalpindi 46000, Pakistan; 7 Department of Environmental Sciences, Kohsar University Murree, Murree 47150, Pakistan; 8 Pediatric Senior Registrar, King Salman Armed Forces Hospital in Northwestern Region (KSAFH), Tabuk 47512, Saudi Arabia; 9 Bioinformatic Department, Genetic Engineering and Biotechnology Research Institute, University of Sadat City, Sadat City 32897, Egypt

**Keywords:** SARS-CoV-2, acid sphingomyelinase (ASM), functional inhibitors, molecular docking, molecular dynamics, COVID-19

## Abstract

Over the past few years, COVID-19 has caused widespread suffering worldwide. There is great research potential in this domain and it is also necessary. The main objective of this study was to identify potential inhibitors against acid sphingomyelinase (ASM) in order to prevent coronavirus infection. Experimental studies revealed that SARS-CoV-2 causes activation of the acid sphingomyelinase/ceramide pathway, which in turn facilitates the viral entry into the cells. The objective was to inhibit acid sphingomyelinase activity in order to prevent the cells from SARS-CoV-2 infection. Previous studies have reported functional inhibitors against ASM (FIASMAs). These inhibitors can be exploited to block the entry of SARS-CoV-2 into the cells. To achieve our objective, a drug library containing 257 functional inhibitors of ASM was constructed. Computational molecular docking was applied to dock the library against the target protein (PDB: 5I81). The potential binding site of the target protein was identified through structural alignment with the known binding pocket of a protein with a similar function. AutoDock Vina was used to carry out the docking steps. The docking results were analyzed and the inhibitors were screened based on their binding affinity scores and ADME properties. Among the 257 functional inhibitors, Dutasteride, Cepharanthine, and Zafirlukast presented the lowest binding affinity scores of −9.7, −9.6, and −9.5 kcal/mol, respectively. Furthermore, computational ADME analysis of these results revealed Cepharanthine and Zafirlukast to have non-toxic properties. To further validate these findings, the top two inhibitors in complex with the target protein were subjected to molecular dynamic simulations at 100 ns. The molecular interactions and stability of these compounds revealed that these inhibitors could be a promising tool for inhibiting SARS-CoV-2 infection.

## 1. Introduction

Coronavirus disease (COVID-19) first emerged in Wuhan, China, in 2019 [1] and later spread worldwide, becoming a global health emergency in 2020 [2]. SARS-CoV-2, a pathogenic virus responsible for COVID-19 spreads primarily through droplets of saliva or discharge from the nose when an infected person coughs or sneezes. It mostly causes respiratory tract infections [3] and is a major therapeutic problem that requires proper care and treatment [4]. Studies suggest that the prevalence of depression significantly increased during the pandemic, with people reporting more symptoms of depression and anxiety than before [5]. Therefore, there is an urgent need to develop an antiviral drug in order to treat this infection and prevent its entry, replication, and transmission [6].

Numerous studies have reported the role of lipids in viral infections, including the life cycle of hepatitis C virus (HCV) [7,8]. Lipid metabolism is also involved in multiple stages of influenza A virus replication and can be a potential target for future IAV drugs [9]. These drugs modulate the viral life cycle, including fusion of the viral membrane to the host cell, viral replication, viral endocytosis, and exocytosis [8]. In COVID-19, lipids are not only strongly altered, but also play a significant role in the pathogenesis and progression of SARS-CoV-2 infection. Lipids, as key regulators in the life cycle of viruses, act as receptors for viral attachment and membrane fusion [10,11]. Sphingolipids are the class of lipids that have a significant impact on viral infection, including their entry, replication, and exit from the host cell. Several viruses such as human immunodeficiency virus (HIV), rhinovirus, Ebola virus, and measles virus use specific sphingolipids for entry into the host cell [12]. Glycosphingolipids such as galactosylceramide serve as receptors for various viruses [10]. 

Sphingolipids [13] have also been reported to modulate SARS-CoV-2 infection [14]. Acid Sphingomyelinase (ASM) is an essential lipid metabolizing enzyme that plays a crucial role in various pathogenic infections, including Neisseria gonorrhoeae, HIV-I, and SARS-CoV-2 [15,16]. ASM functions by cleaving sphingomyelin, a type of sphingolipid, to ceramide, thus mediating viral entry into the cell [14]. This is cohesive with a recent study that reported increased levels of ASM and ceramide and decreased levels of sphingomyelin in patients with severe COVID-19 [14]. Therefore, it has been confirmed that ASM inhibition can potentially reduce SARS-CoV-2 entry into the cells. ASM catalyzes the conversion of sphingomyelin into ceramide [17], forming gel-like platforms in the plasma membrane that are further utilized by SARS-CoV-2 for entry into the cells. A study by Carpinteiro et al. [14] reported that the acid sphingomyelinase/ceramide system plays an important role in SARS-CoV-2 infection [14]. The authors demonstrated that the functional inhibition of the ASM/ceramide system with antidepressants reduced the infection with authentic/pseudo viral SARS-CoV-2 in vitro. Therefore, lowering the amount of ceramide through the downregulation/inhibition of ASM can be a therapeutic target for protection against SARS-CoV-2 infection [18]. 

Multiple studies [16,19] have suggested the significance of FIASMAs (functional inhibitors against ASM) for repurposing as potential drugs against COVID-19. One such anti-depressant, namely fluoxetine, has been tested in vitro for the inhibition of ASM [20]. Various FIASMAs have been identified through in silico, in vitro, or in vivo studies as being potential antiviral drug candidates against SARS-CoV, MERS-CoV, and SARS-CoV-2 [6,21]. Additionally, a study of hospitalized adults illustrated that patients who were given FIASMA medications were less likely to be intubated or die [22]. As targeting SARS-CoV-2 viral entry can serve as an anti-viral therapy, a wide variety of FIASMAs can be used potential anti-virals for host-directed therapy and can be repurposed as anti-viral drugs against SARS-CoV-2 [23].

Various therapeutic targets have been reported for COVID-19, including spike protein (S-RBD), IL-6 [24], ACE-2 [25,26], and COVID-19 main protease (MPro) [27,28]. A number of in silico studies including virtual screening and molecular docking have been performed with these targets in an effort to restrict SARS-CoV-2 infection [22,23]. Hitherto, to the best of our knowledge, no in silico research has been carried out where ASM was targeted to control the infection.

**Figure 1:**
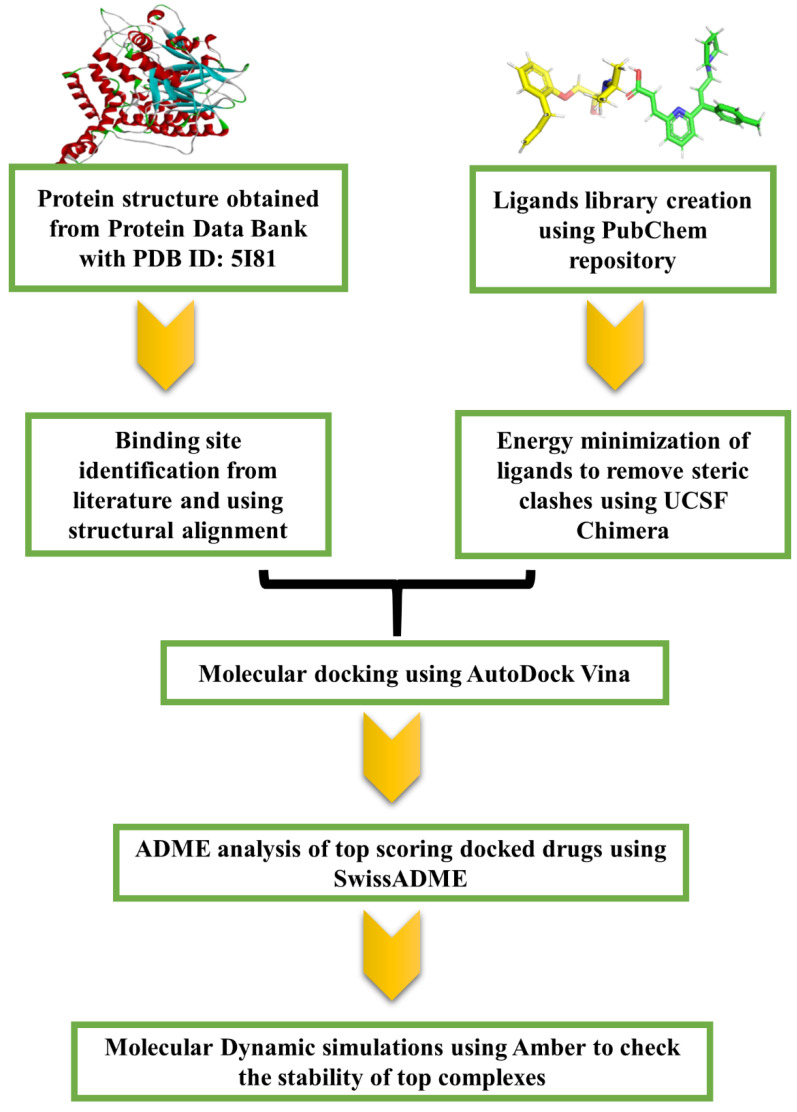
Workflow followed in the present study to identify potential drugs to prevent SARS-CoV-2 infection.

All of the above-mentioned studies showcase the importance of the ASM/ceramide pathway in COVID-19, specifically the inhibition of the acid sphingomyelinase (ASM) enzyme. As reported some anti-depressants are widely used in clinical practice and have a favorable safety profile, there is thus a need to repurpose these FIASMAs against ASM for SARS-CoV-2 infection. The present study was aimed towards achieving the aforementioned goal. To effectuate the inhibitor library against acid sphingomyelinase (ASM) was generated followed by computational molecular docking of the potential inhibitors with the target protein, i.e., ASM. The docking results and ADME properties were analyzed and the top two potential drugs were subjected to molecular dynamics simulation analysis. The workflow of the present study is demonstrated in Figure 1. These inhibitors should be further tested experimentally for approval by the FDA.

## 2. Results

### 2.1. Target Protein Structure 

**Figure 2:**
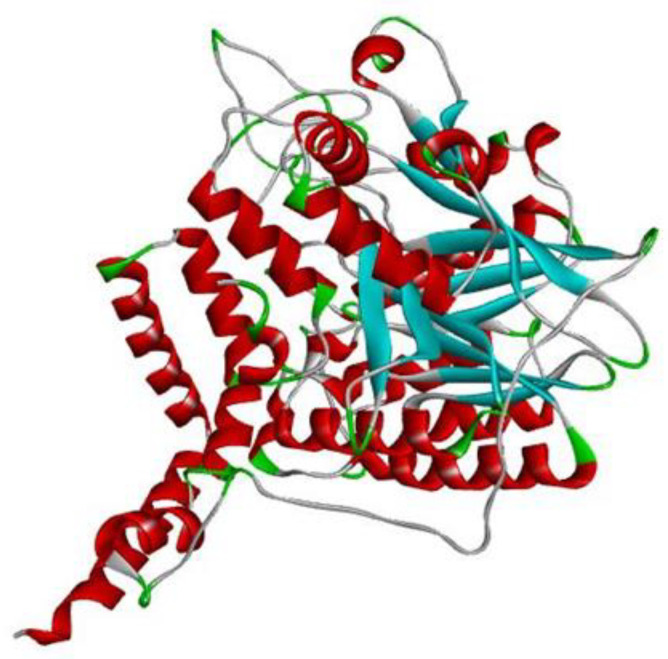
Discovery studio visualizer representation of the PDB structure of human acid sphingomyelinase, i.e., ASM with zinc; EC number-3.1.4.12; PDB ID-5I81. Red represents helices, blue indicates beta sheets and grey corresponds to loop regions.

The target protein human acid sphingomyelinase, i.e., ASM, with zinc (PDB ID: 5I81) [29], for which no computational work has been reported up until now, is presented in Figure 2.

Human acid sphingomyelinase (ASM) with zinc (PDB ID: 5I81) has been classified as a hydrolase [30]. ASM catalyzes the hydrolysis of sphingomyelin to ceramide and phosphocholine [29]. The enzyme plays a key role in the signaling pathways mediated by ceramide [29]. After visualizing the protein in Discovery Studio visualizer, it was found that human acid sphingomyelinase consists of only one chain and that one chain is involved in the interaction with FIASMAs [29]. 

### 2.2. Binding Site Prediction

**Figure 3:**
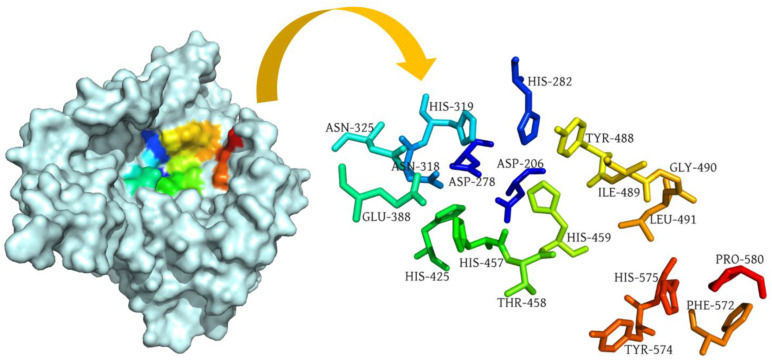
Surface representation of human acid sphingomyelinase (PDB: 5I81) with labelled binding pocket residues demonstrated in rainbow colors.

Sequence alignment analysis yielded 86% sequence similarity between human acid sphingomyelinase (PDB: 5I81) and murine acid sphingomyelinase bound with the inhibitor AbPA (PDB: 5FI9), which suggests that murine ASM can be used as a reference protein to predict the potential binding pocket of our target protein, i.e., human ASM. Human ASM consists of one chain, i.e., chain A, whereas murine ASM possesses two chains, i.e., chain A and chain B. Both proteins were structurally aligned using PyMol and the alignment indicated that chain B of the reference protein (murine ASM) aligned with chain A of the target protein. Following the structure alignment, the binding pocket residues of the murine ASM were used as a reference to predict the pocket residues of the target protein. The binding pocket of human ASM is shown in Figure 3. The histidine at position 459 served as a central residue of the binding pocket.

### 2.3. Molecular Docking and ADME Analysis

**Figure 4:**
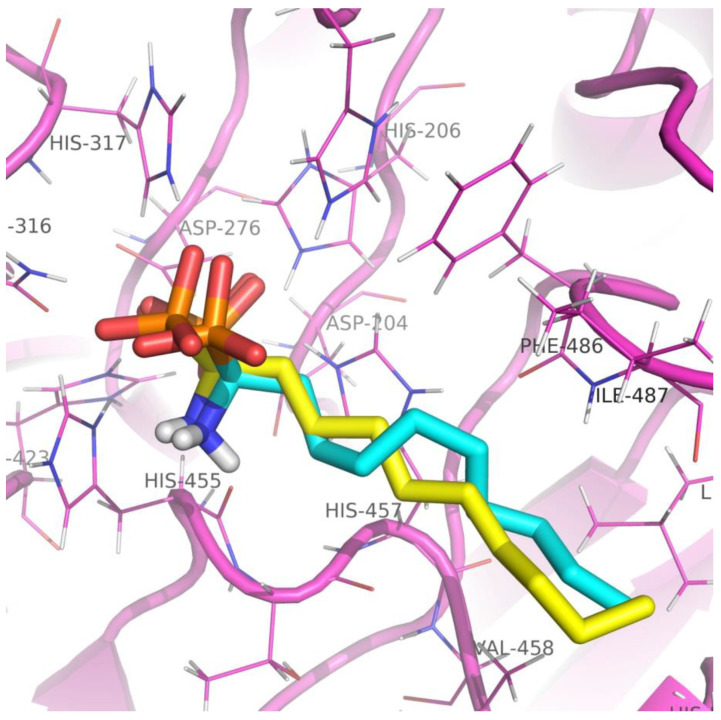
Three-dimensional representation of the native AbPA ligand (yellow) and docked AbPA ligand (blue) in the crystal structure of murine acid sphingomyelinase. The protein with active site residues is displayed in pink.

Before carrying out the docking of FIASMAs with the target protein, the docking protocol was applied on murine ASM (PDB: 5FI9), where the native ligand AbPA was docked with the crystal structure. Multiple binding poses were generated and subjected to RMSD analysis. It was observed that the top scoring docked pose exhibited a minimum RMSD of 1.01 Å when compared with the native ligand (Figure 4). The outcome validated that the designed docking protocol and docking experiments were carried out with FIASMAs and human ASM. The docking experiments revealed strong interactions of FIASMAs with the target protein (human ASM). Various binding modes were recorded, each with a specific binding affinity. The binding mode with the least binding affinity was regarded as the best mode because of its stability [31]. The top 5% drug complexes were shortlisted based on the binding affinity scores. Table 1 depicts the details of the binding affinity scores and ADME analysis details, including the molecular weight, XlogP value, number of H-bond donors and acceptors, polar surface area, number of rotatable bonds, and Lipinski violation for the top 5% docked drugs. The binding scores for all of the inhibitors is provided in supplementary Table S1. The ADME analysis revealed that the top three drugs with the lowest binding affinity that were also non-toxic were Cepharanthine, Zafirlukast, and Atovaquone.

The interactions of the specific amino acids taking part in the binding of the drug and protein were also recorded. The docked complexes were visualized in Discovery Studio.

### 2.4. Visualization and Analysis of Docked Complexes

**Figure 5:**
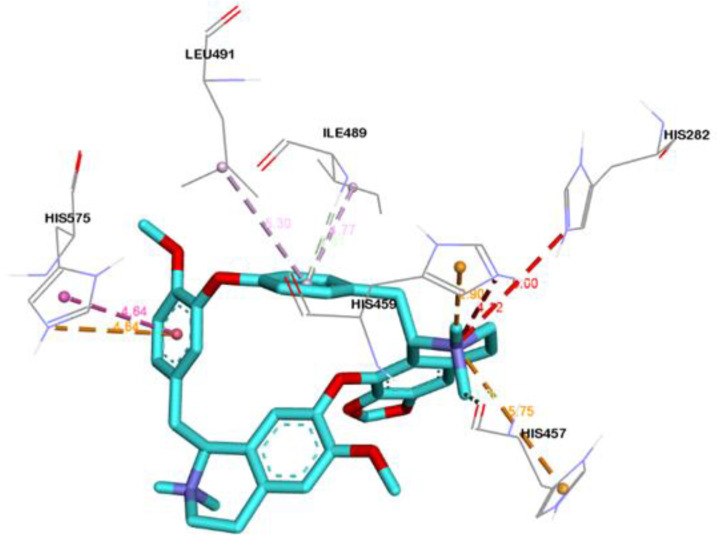
Three-dimensional interaction diagram showing the molecular interactions of Cepharanthine with binding pocket residues (grey). Molecular interactions include carbon hydrogen bond (pale green), unfavorable positive–positive (red), pi–cation (tangerine), pi–donor hydrogen bond (transparent green), pi–pi T-shaped (rose pink), and alkyl and pi–alkyl (light pink).

**Figure 6:**
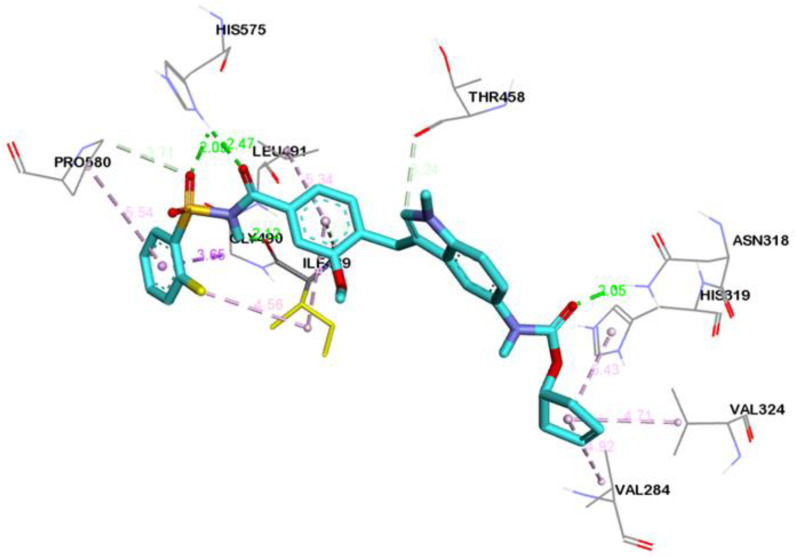
3D interaction diagram showing molecular interactions of Zafirlukast with the amino acids (grey) mainly involved in the binding pocket identified. Molecular interactions include conventional hydrogen bond (green), carbon–hydrogen bond and pi–donor hydrogen bond (pale green), pi–sigma (purple), and alkyl and pi–alkyl (light pink).

**Figure 7:**
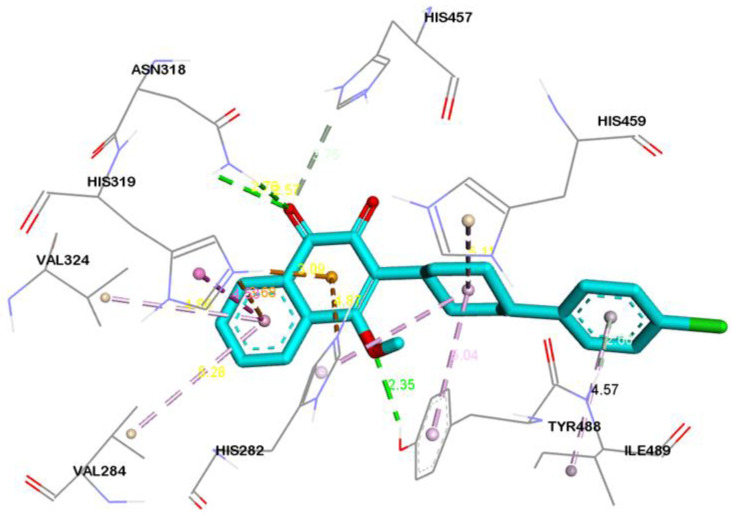
3D interaction diagram showing molecular interactions of Atovaquone with the amino acids (grey) mainly involved in the binding pocket identified. Molecular interactions include conventional hydrogen bond (green), carbon hydrogen and pi–donor hydrogen bond (pale green), pi–cation (tangerine), pi–pi T-shaped (rose pink), and alkyl and pi–alkyl (light pink).

Three-dimensional view of the receptor−ligand interactions of the best poses generated by the top three ligands, i.e., Cepharanthine, Zafirlukast, and Atovaquone, are shown in Figure 5, Figure 6, and Figure 7, respectively. The amino acid residues in the binding site are shown in the form of thin grey colored lines, while the ligand itself is represented in blue sticks. Interactions such as hydrogen bonds and pi−pi interactions are shown in dotted lines. The graphical visualization was performed on Discover Studio visualizer and images were recorded at an optimal viewing angle that best described the protein–ligand interactions. Details of the active site residues involved in various interactions along with bond distances are tabulated in Table 2. 

Cepharanthine docked with human ASM showed significant binding, yielding a binding affinity of −9.6 kcal/mol (Figure 5) and is considered as a top scoring drug. The ligand Cepharanthine was predicted to be bound to the inside of the binding pocket forming eight molecular interactions with the central residue of the binding pocket, i.e., HIS-459 and key amino acids of the binding pocket of target protein (Figure 5). The major interactions between Cepharanthine and binding pocket residues of human ASM include hydrogen bonds between Cepharanthine HIS-459, pi–pi interaction with HIS-575, alkyl interactions with ILE-489 and LEU-491, etc. In the ADME analysis, Cepharanthine was found to obey Lipinski’s rule with log *p* value of 6.5, no hydrogen bond donors, 8 hydrogen bond acceptors, a topological surface area of 61.9 Å^2^, 2 rotatable bonds, and 1 violation, i.e., molecular weight greater than 500.

The docking of Zafirlukast with human ASM revealed that the ligand had a high affinity of −9.5 kcal/mol. Molecular docking of Zafirlukast with the target protein led to 15 molecular interactions between the ligand and the pocket residues of human ASM. The best fitting of ligand inside the core pocket region of ASM was further evidenced by the conventional hydrogen bond of Zafirlukast with ASN-318, HIS-575, and ILE-489, and pi–alkyl and alkyl interaction with ILE-489 (Figure 6). On ADME analysis, Zafirlukast was found obeying Lipinski’s rule with log *p* value of 5.5, 2 hydrogen bond donors, 6 hydrogen bond acceptors, topological surface area of 54.4 Å^2^, 2 rotatable bonds and 1 violation i.e., molecular weight greater than 500.

With three conventional hydrogen bonds—one carbon–hydrogen bond and two pi–donor hydrogen bonds—Atovaquone showed promising activity with human ASM with binding affinity of −9.1 kcal/mol. It showed strong interaction with human ASM in the binding pocket (Figure 7). The major interactions between the ligand and binding pocket residues of the target protein include the conventional hydrogen bond of Atovaquone with ASN-318 and TYR-488, the carbon–hydrogen bond with HIS-457, pi–cation interaction with HIS-319, and alkyl interaction with LEU-491. In the ADME analysis, Atovaquone was found to be obeying Lipinski’s rule with a log *p* value of 5.2, one hydrogen bond donors, three hydrogen bond acceptors, topological surface area of 54.4 Å^2^, two rotatable bonds, and a molecular weight of 366.8.

These docking results indicate that the top FIASMAS under consideration, namely Cepharanthine, Zafirlukast, and Atovaquone, strongly bind to human acid sphingomyelinase. The FIASMAS established strong molecular interactions and thus can effectively be used to inhibit ASM, thereby inhibiting SARS-CoV-2 entry into the cell. Furthermore, these results emphasize the need for the adaptation of docking-based drug discovery approaches for other target proteins that are pharmacologically important. 

### 2.5. Molecular Dynamics and Simulation

Based on the docking scores and interactions with the pocket residues, the top two docked complexes, i.e., Cepharanthine docked with target protein (compex-1) and Zafirlukast docked with target protein (complex-2), were subjected to MD simulations to further validate the findings. RMSD and RMSF analyses were carried out to evaluate the stability of the ligand inside binding pocket of the target protein. The complex stability was inferred from three parameters, i.e., fluctuations in the target protein structure, ligand stability inside the binding pocket, and overall deviation in the protein–ligand complex. The RMSD and RMSF values are summarized in Table 3.

**Figure 8:**
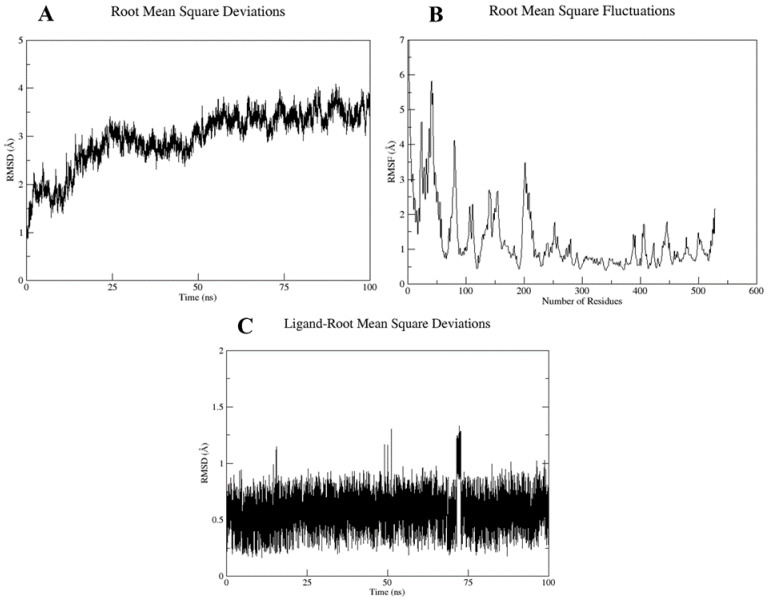
(**A**) Protein–ligand RMSD, (**B**) RMSF versus residue number plot, and (**C**) ligand RMSD for the Complex 1 over the simulation run of 100 ns.

The RMSD plot of complex-1 showed that the protein attained equilibrium after 2.5 ns and remained stable until 100 ns, with minor deviations, with an average RMSD value of 2.9 Å (Figure 8A). RMSF analysis was conducted to explore the per residue fluctuation of the system. The average RMSF recorded was 1.27 Å. Residues at the N-terminal displayed more fluctuations; however, the binding pocket residues were stable (Figure 8B). The ligand RMSD analysis of complex-1 was quite stable with an average RMSD of 0.57 Å, which indicated a stable interaction with the target binding pocket (Figure 8C).

**Figure 9:**
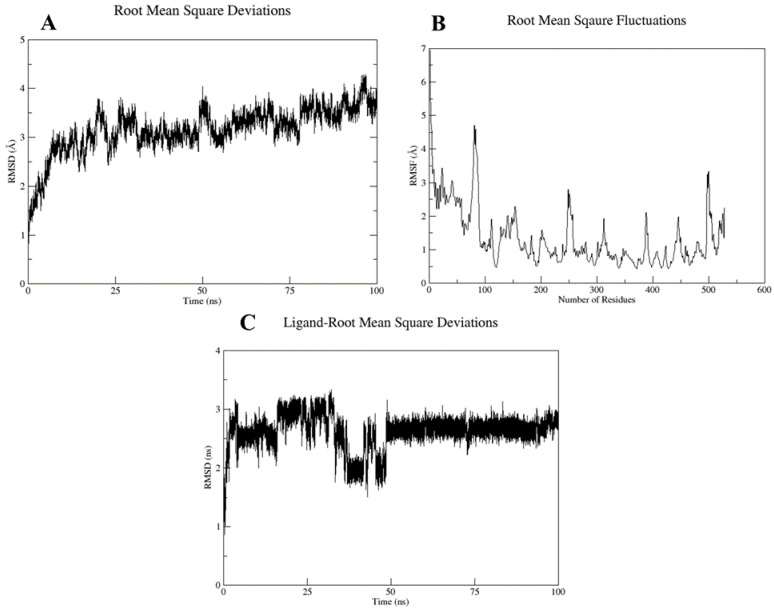
(**A**) Protein–ligand RMSD, (**B**) RMSF versus residue number plot, (**C**) ligand RMSD for the complex-2 over the simulation run of 100 ns.

For complex-2, the average RMSD was 3.17 Å, which also suggests backbone stability. The RMSF value was 1.27 Å; however, the ligand displayed major deviations inside the binding pocket with an average RMSD of 2.62 Å (Figure 9). A high ligand RMSD could be related to the ligand structure, which was more flexible because of the presence of rotatable bonds compared with the complex-1 ligand, which possessed fewer rotatable bonds.

**Figure 10:**
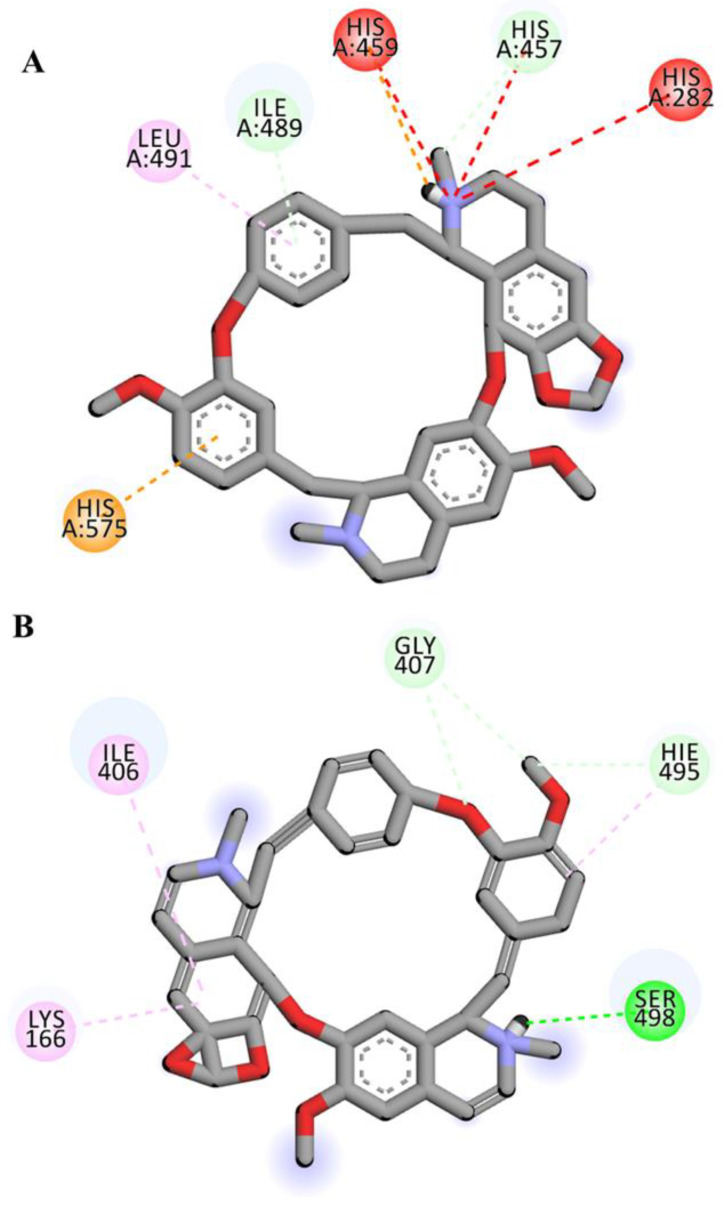
2D representation of complex-1 before (**A**) and after (**B**) the simulation run of 100 ns. The ligand has been colored by element where grey represents carbon atoms, red corresponds to oxygen atoms and purple shows nitrogen atoms.

For complex-1, the receptor–ligand docking interactions displayed multiple interactions, including carbon–hydrogen, pi–cation, pi–donor hydrogen bond, pi–pi, alkyl, pi–alkyl and a few unfavorable interactions (Figure 10A). However, when the receptor–ligand interactions were observed after simulations of 100 ns, SER-498 formed a new conventional hydrogen bond with the ligand, while the carbon–hydrogen and pi–alkyl bonds also remained intact. The unfavorable interactions were also removed following the simulation (Figure 10B).

**Figure 11:**
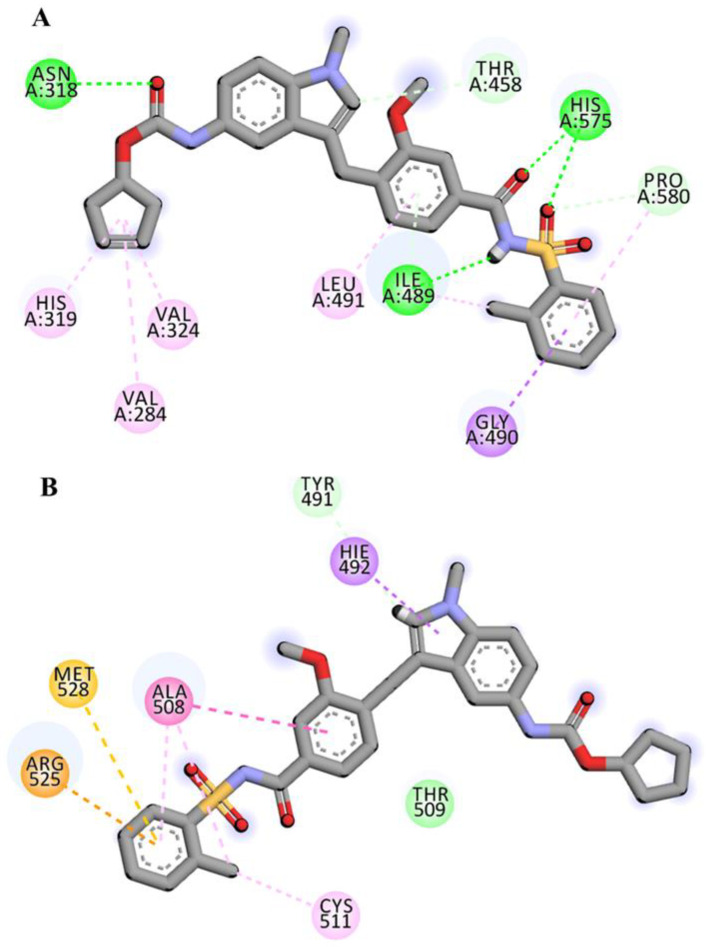
2D representation of complex-2 before (**A**) and after (**B**) the simulation run of 100 ns. The ligand has been colored by element where grey represents carbon atoms, red corresponds to oxygen atoms, purple shows nitrogen atoms and yellow indicates sulfur atom.

For complex-2, the docking interactions showed that the ligand formed strong conventional hydrogen, pi–sigma, and alkyl and pi–alkyl bonds, as well as a carbon hydrogen and pi–donor hydrogen bond with the receptor (Figure 11A). However, when receptor–ligand interactions were observed after 100 ns simulations, the system for complex-2 showed major deviations. The ligand formed van der Waals, carbon–hydrogen, pi–carbon, pi–sigma, pi–sulfur, pi–pi Y shaped, and amide–pi stacked bonds, while the alkyl and pi–alkyl bonds remained intact (Figure 11B).

The aforementioned results suggest that both inhibitors established strong interactions with the binding pocket of human ASM, and thus could serve as potential drugs to inactivate the ceramide pathway, thereby inhibiting viral entry into the host cells. However, the two drugs could further be validated via experimental analysis to confirm the findings.

## 3. Discussion

Carpinteiro et. al. [14] found out the ASM/ceramide system was activated by SARS-CoV-2, resulting in the formation of ceramide-enriched membrane domains, which facilitated viral entry and infection by forming cluster-like structures with the ACE2 receptor. Acid sphingomyelinase (ASM) is an enzyme that is responsible for cleaving sphingomyelin to ceramide [32]. Therefore, inhibiting ASM will lead to viral blockage [14]. As ASM is found in intralysosomal membranes, it is protected from proteolytic inactivation. The most efficient way is to functionally inhibit ASM. FIASMAs also known as functional inhibitors of acid sphingomyelinase are the group of drugs that could functionally inhibit ASM [16,19]. FIASMAs have been reported to reduce the activity of ASM in human patients [33,34,35]. In present study we employed computational techniques to report that blockade or downregulation of ASM with FIASMAs will protect against the infection with SARS-CoV-2. Studies have reported multiple receptors including angiotensin-converting enzyme (ACE2), dipeptidyl peptidase-4 (DPP4) and glucose-regulating protein 78 (GRP78) that are used by virus to translocate into the cells [36]. In this study, we have specifically addressed ACE2 receptor to curb the COVID-19 by targeting ASM pathway. Recently various mutations have been reported in SARS-CoV-2 variants including K417N, K417T, N501Y, E484K, and S477N. These mutations lead to an increased affinity for ACE2 [37]. This indicates that ACE2 is the most common receptor used by SARS-CoV-2 for infection and blocking it via targeting ASM is a promising strategy to control the viral infection.

With aim to discover new potential inhibitor for SARS-CoV-2, we repurposed FIASMAs inhibiting human ASM against COVID-19. Encouraged by earlier studies [22], we have utilized in-silico techniques to unravel the potential of FIASMAs against ASM to target SARS-CoV-2. Library of FIASMAs as reported in studies [16,19] was constructed. Three dimensional molecule structures were downloaded from PUBCHEM followed by the energy minimization to relax the steric clashes if any. To the best of our knowledge, this is the first study where molecular computational techniques are applied to screen FIASMAs against human acid sphingomyelinase to inhibit the infection by SARS-CoV-2. Total 258 FIASMAs were docked with human ASM. Molecular docking generated binding affinity scores for all the docked complexes. An effective drug must not only bind strongly with the target but also have appropriate ADME properties [38]. Although most of the FIASMAs possess favorable ADME properties but to rule out any possibility the top 5% of the best scoring docked complexes were further tested for ADME properties using SwissADME [39]. SwissADME computes physicochemical descriptors, ADME and pharmacokinetic properties. These properties help in deducing the drug-like nature of molecules. Based on binding scores and ADME properties, three potential drugs were shortlisted namely Cepharanthine, Zafirlukast, Atovaquone with the score of −9.6, −9.5 and −9.1 kcal/mol respectively. It has been observed that all three drugs accommodated at the binding pocket of human ASM, established key interactions with the pocket residues. Cepharanthine formed multiple carbon hydrogen bonds with key residues of binding pocket including HIS-457, ILE-489. The role of hydrogen bond is well established in intermolecular interactions [40]. Although CH bonds has received less importance in the process of drug design, their potential significance cannot be ignored [41,42]. Earlier studies have reported the significance of CH bonds in molecular recognition [43,44,45]. In addition, the ligand also displayed hydrophobic interactions with the binding pocket of human ASM (Figure 5).

Zafirlukast established strong interactions with vital residues of target protein. These include four conventional hydrogen bonds within distance of 3 Å, two carbon-hydrogen bonds at distance of 3.2 Å and 3.7 Å and one pi-donor hydrogen bond at 2.6 Å. Besides hydrogen bonds, multiple alkyl, pi-alkyl and pi-sigma bonds were also evident (Figure 6). All of these mentioned interactions are in the vicinity of the active site region. Atovaquone the third top scoring drug formed three conventional hydrogen bonds, one carbon-hydrogen bond, two pi-donor hydrogen bonds, and multiple pi-alkyl (hydrophobic) and pi-cation (electrostatic) interactions with the active site region of target protein (Figure 7). 

To further validate the stability of these interactions, the top two docked complexes were subjected to molecular dynamic simulation of 100 ns. The simulation analysis demonstrated that the top scoring Cepharanthine retained the position at the binding site with slight variations in molecular interactions. For complex-1 (Cepharanthine), two unfavorable interactions were removed that were present before the simulation. One new hydrogen bond was established between the potential drug and SER-498 of the target protein. Two carbon hydrogen bonds were formed with the pocket residue GLY-407 (Figure 10B). In addition to the hydrogen bonds, hydrophobic interactions were also observed. Hydrophobic interactions are crucial to the binding and specificity of drug molecules with biomolecules, and play a significant role in enhancing the inhibitor affinity and selectivity [46,47]. Cepharanthine exhibited two hydrophobic (alkyl) interactions with LYS-166 and ILE-406, thereby enhancing the stability of complex-1, as also evident from the RMSD of the complex (2.99 Å) and ligand RMSD (0.57 Å) (Figure 8).

In the case of complex-2 (Zafirlukast), more variations were recorded following the simulation of 100 ns. Before the simulation, the drug was well accommodated in the binding pocket, displaying strong interactions with the residues (Figure 11A). However, after the simulation, hydrogen bonds were removed and the number of hydrophobic interactions were reduced as well (Figure 11). The RMSD of the ligand inside the pocket was recorded at 2.62 Å, which suggested the ligand was slightly unstable compared with the first complex. 

Jeon et al. [48] also reported an antiviral activity of Cepharanthine against SARS-CoV-2 in Vero cells. The computational findings from our study also provide significant evidence in favor of the mentioned drug. The simulation RMSD graphs of the complex and ligand and the RMSF graph of the protein backbone further conferred the stability of protein–ligand complex, making it a plausible inhibitor. This compound can further be tested experimentally and used as an initial hit to develop new inhibitors.

## 4. Materials and Methods

### 4.1. Target Protein Selection and Preparation 

The 3D structure of human acid sphingomyelinase (ASM) was retrieved from the RCSB Protein Databank [49] (PDB ID: 5I81) [29]. The protein was preprocessed using AutoDock Tools [50], i.e., polar hydrogens were added and water molecules and HETATMS were removed from the pdb file. The structure was saved in pdbqt format for docking.

### 4.2. Binding Site Identification

Prior knowledge of the binding site is important for carrying out computational docking. As the binding pocket of human ASM has not yet been reported, we predicted the site through structure alignment with another protein (PDB: 5FI9) [51], having known the binding site that shared 80% sequence similarity with the target protein. Furthermore, 5FI9 is the closed-form of murine acid sphingomyelinase in complex with bisphosphonate inhibitor AbPA. Here, 5FI9 served as a reference protein to find the potential binding site of the target protein, as both the proteins have similar functions, i.e., both convert sphingomyelin to ceramide and belong to the class of enzymes, i.e., hydrolases. These proteins differ based on the organism in which they are present, i.e., humans and mice for 5I81 and 5FI9, respectively.

ClustalW [52] was used for performing the sequence alignment and PyMol [10] was used to carry out the structural alignment of both the proteins.

### 4.3. Ligand Collection and Preparation

Previous studies have reported functional inhibitors against ASM (FIASMAs) [14]. These inhibitors were exploited to block the entry of SARS-CoV-2 into cells. A small drug library was prepared by retrieving the 3D structure of FIASMAS from the PubChem Database [46]. The collected inhibitors were minimized using USCF Chimera [53] to remove any steric clashes. OpenBabel [54] was employed to convert the ligands to pdbqt format for the docking run.

### 4.4. Molecular Docking

For Molecular Docking [55], AutoDock Vina [51] was used. Water molecules and HETATMS were removed from the crystal structure prior to the docking. Grid Box [56] was set as per the predicted binding site using 5IF9 as the reference protein. The grid box size was set to 30 Angstroms, while the values of the x, y, and z coordinates of the grid center were set to -11.031, −30.163, and −28.533, respectively. Exhaustiveness and number of modes were set to 16 and 20, respectively. The receptor file was saved in pdbqt format. The Linux operating system was used with three processors and 4 GB RAM in a virtual box. It took approximately 1–2 min for one ligand to dock to the receptor molecule, while the overall time for 257 drugs was approximately 3–4 h. As a retrospective control, the molecular docking of AbPA to the murine acid sphingomyelinase (PDB: 5FI9) was also carried out using the same software and parameters. RMSD of the docked ligand pose with native pose was calculated using DockRMSD tool [57].

### 4.5. ADME Analysis

As the early estimation of ADME properties can greatly reduce the chances of pharmacokinetics-related failure in the clinical phases [58], the docking results were screened based on the binding affinity scores as well as ADME properties [38,59]. In silico screening of the pharmacological properties ADME and evaluation of drug-likeness were performed using the SwissADME web tool [39] and PubChem database [60]. The drug-likeness prediction was based on several rules, including Lipinski [61], Ghose, Veber, Egan, and Mugge [62]. The pharmacokinetics properties observed were: molecular weight, XLogP3-AA, H-bond Donor, H-bond Acceptor, Topological polar surface area, No. of rotatable bonds.

### 4.6. Molecular Dynamic Simulation

Here, 100 ns MD simulation was carried out using AMBER 20 software for the selected docking complexes [63] using a similar protocol, as defined in a previous study [64]. Ligand preparation was carried out using the Antechamber program [65] in the Amber12 tools. The Amber forcefield (GAFF) was chosen for the ligand [66], whereas the forcefield 99SB was used for the protein. The complex integration into a TIP3P water box was accomplished where the padding distance was set to 12 Å between the protein and box boundaries. Neutralization of the system was achieved through the addition of Na^+^ ions to it. The system hydrogen atoms, solvation box, carbon alpha atoms, and all non-heavy atoms were minimized for 500, 1000, 1000, and 300 steps, respectively. Subsequently, heating of the system to 300 K (NVT) for 20-ps was done in langevin dynamics to maintain system temperature. Here, restraint of 5 kcal/mol-A2 on carbon alpha atoms at a time step of 2-fs was allowed. In equilibration, the system was relaxed for 100-ps. System pressure was maintained by means of NPT ensemble for 50 ps. While preparing the system for simulation, the protein was numbered from position 1, unlike the protein available in crystal form (PDB: 5I81), which started from position 84. This led to variations in the position numbering of amino acids in the simulated complex. The simulation trajectories were further assessed by analyzing the various physical properties: Root Mean Square Deviation (RMSD) of the complex molecule, ligand RMSD, and root mean square fluctuation (RMSF).

## 5. Conclusions

Herein, we targeted acid sphingomyelinase (ASM) to prevent SARS-CoV-2 infection. Studies have indicated that ASM converts sphingomyelin into ceramide and thus facilitates viral entry into the cell. Therefore, if acid sphingomyelinase activity is inhibited pharmacologically or surface ceramide is neutralized, SARS-CoV-2 infection in the cells will be prevented. A library of reported functional inhibitors against ASM (FIASMAs) was constructed from PubChem. These inhibitors were docked computationally with human ASM. The idea was to repurpose these inhibitors to block the entry of SARS-CoV-2 into cells. The docking results revealed strong interactions of these inhibitors with human ASM, making them potential drugs against SARS-CoV-2. The top 5% of the docked inhibitors were subjected to ADME analysis and the results were further screened based on the toxic and non-toxic properties. Together, docking binding affinity scores and ADME properties analysis demonstrated that the top three drugs with the lowest binding affinity and those that were non-toxic were Cepharanthine, Zafirlukast, and Atovaquone. These drugs established strong molecular interactions with the target protein and can effectively be used to inhibit ASM, thereby inhibiting SARS-CoV-2 entry into the cell. To validate it further, the top two complexes were subjected to molecular dynamic simulations for a time period of 100 ns. The simulation analysis revealed that between the two, Cepharanthine displayed an overall higher stability inside the binding pocket compared with to Zafirlukast. Therefore, this drug can serve as a plausible drug to control SARS-CoV-2 infection. This study is a key step towards further exploiting the ASM inhibitors for controlling COVID-19 and designing new drugs targeting SARS-CoV-2.

## Figures and Tables

**Table 1:** Table representing the top 5% drugs selected after docking (on the basis of binding affinities) and ADME toxicity analysis.

Ligands	Binding Affinity	Molecular Weight	XLogP3-AA	H-Bond Donor	H-Bond Acceptor	Topological Polar Surface Area (Å^2^)	No. of Rotatable Bonds	Lipinski Violation
Dutasteride	−9.7	528.5	5.4	2	8	58.2	2	No-2
Cepharanthine	−9.6	606.7	6.5	0	8	61.9	2	Yes-1
Zafirlukast	−9.5	575.5	5.5	2	6	124	9	Yes-1
Carbenoxolone	−9.2	570.8	6.4	2	7	118	6	No-2
Telmisartan	−9.2	514.6	6.9	1	4	72.9	7	No-2
Atovaquone	−9.1	366.8	5.2	1	3	54.4	2	Yes-0
Doxorubicin	−8.9	543.5	1.3	6	12	206	5	No-3
Pirarubicin	−8.9	627.6	2.7	5	13	204	7	No-2
Profenamine	−8.7	312.5	4.8	0	3	31.8	5	Yes-1
Ritanserin	−8.7	477.6	5.2	0	6	61.2	5	Yes-1
Solasodine	−8.7	413.6	5.4	2	3	41.5	0	Yes-1
Tomatidine	−8.7	415.7	6.2	2	3	41.5	0	Yes-1
Astemizole	−8.6	458.57	5.97	4	1	42.32	8	Yes-1

**Table 2:** Table displaying docking interactions and bond distances of the top scoring ligands with the active site of the target protein.

Ligand Name	Residues Involved in Interaction	Type of Interaction	Bond Distance (Å)
Cepharantine	LIG:C---HIS457:O	Carbon Hydrogen Bond	2.99
	HIS575:NE2---LIG	Pi-Cation	4.64
	LIG:H---HIS459	Pi-Cation; Pi-Donor Hydrogen Bond	2.89
	ILE489:HN---LIG	Pi-Donor Hydrogen Bond	2.60
	HIS575---LIG	Pi-Pi T-shaped	4.64
	LIG:C---PRO580	Alkyl	3.65
	HIS430---LIG:C	Pi-Alkyl	5.01
	HIS575---LIG:C	Pi-Alkyl	4.64
	LIG---ILE489	Pi-Alkyl	4.76
	LIG---LEU491	Pi-Alkyl	5.30
Zafirlukast	ASN318:HD21---LIG:O	Hydrogen Bond	2.05
	HIS575:HD1---LIG:O	Hydrogen Bond	2.46
	HIS575:HD1---LIG:O	Hydrogen Bond	2.09
	LIG:H---ILE489:O	Hydrogen Bond	2.11
	PRO580:CD---LIG:O	Carbon Hydrogen Bond	3.70
	LIG:C---THR458:O	Carbon Hydrogen Bond	3.23
	ILE489:HN---LIG	Pi-Donor Hydrogen Bond	2.61
	GLY490:CA---LIG	Pi-Sigma	3.64
	VAL284---LIG	Alkyl	4.91
	VAL324---LIG	Alkyl	4.70
	LIG:C---ILE489	Alkyl	4.55
	LIG:C---ILE489	Alkyl	4.81
	HIS319---LIG	Pi-Alkyl	5.42
	LIG--- ILE489	Pi-Alkyl	4.70
	LIG---LEU491	Pi-Alkyl	5.34
Atovaquone	ASN318:HD21---LIG:O	Hydrogen Bond	2.57
	ASN318:HD22---LIG:O	Hydrogen Bond	2.76
	TYR488:HH---LIG:O	Hydrogen Bond	2.35
	HIS457:CE1---LIG:O	Carbon Hydrogen Bond	3.75
	HIS282:NE2---LIG	Pi-Cation	4.87
	HIS319:NE2---LIG	Pi-Cation	3.68
	HIS319:HE2---LIG	Pi-Cation; Pi-Donor Hydrogen Bond	3.08
	ILE489:HN---LIG	Pi-Donor Hydrogen Bond	2.60
	HIS319---LIG	Pi-Pi T-shaped	4.52
	LIG:CL---VAL460	Alkyl	5.40
	LIG:CL---LEU491	Alkyl	4.24
	HIS459---LIG	Pi-Alkyl	5.11
	TYR488---LIG	Pi-Alkyl	5.03
	HIS575---LIG:CL	Pi-Alkyl	4.93
	LIG---VAL284	Pi-Alkyl	5.27
	LIG---VAL324	Pi-Alkyl	4.85
	LIG---ILE489	Pi-Alkyl	4.56

**Table 3:** Root mean square deviation and root mean square fluctuation for the top two complexes subjected to molecular dynamic simulation analysis.

Parameters	Complex 1	Complex 2
RMSD of complex	2.99 Å	3.17 Å
Ligand RMSD	0.57 Å	2.62 Å
RMSF	1.27 Å	1.33 Å

## Data Availability

The raw data supporting the conclusions of this article will be made available by the authors, without undue reservation.

## Supplementary Materials

The following supporting information can be downloaded at: https://www.mdpi.com/article/10.3390/molecules28072989/s1. Table S1: Binding Scores of all inhibitors.
