# Supplementary material for: Repurposing FIASMAs against Acid Sphingomyelinase for COVID-19: A Computational Molecular Docking and Dynamic Simulation Approach
Source: Molecules. 2023 Mar 27;28(7):2989. doi: 10.3390/molecules28072989 (PMC10096053; doi:10.3390/molecules28072989)
Supplement: Supplementary file 1 [file molecules-28-02989-s001.zip › molecules-2281475-supplementary.pdf]

**Supplementary Table S1:** Binding Scores of all inhibitors

| **Drug** | **Affinity (kcal/mol)** |
|---|---|
| Dutasteride | -9.7 |
| Cepharantine | -9.6 |
| Zafirlukast | -9.5 |
| Carbenoxolone | -9.2 |
| Telmisartan | -9.2 |
| Atovaquone | -9.1 |
| Doxorubicin | -8.9 |
| Pirarubicin | -8.9 |
| Profenamine | -8.7 |
| Ritanserin | -8.7 |
| Solasodine | -8.7 |
| Tomatidine | -8.7 |
| Astemizole | -8.6 |
| Daunorubicin | -8.6 |
| Diosmin | -8.6 |
| Droperidol | -8.4 |
| Montelukast | -8.3 |
| Flunarizine | -8.2 |
| SB-222200 | -8.2 |
| Sertindole | -8.1 |
| Trifluperidol | -8.1 |
| Domperidone | -8 |
| Fusidic-acid | -8 |
| Conessine | -7.9 |
| Dexamethasone | -7.9 |
| Donepezil | -7.9 |
| Enoxolone | -7.9 |
| Lercanidipine | -7.9 |
| Raloxifene | -7.9 |
| Spiperone | -7.9 |
| Bromocriptine | -7.8 |
| Calcipotriol | -7.8 |
| Cinnarizine | -7.8 |
| Fipexide | -7.8 |
| Phenserine | -7.8 |
| Pirenperone | -7.8 |

| | |
|---|---|
| Progesterone | -7.8 |
| Clofazimine | -7.7 |
| Desloratadine | -7.7 |
| Flavoxate | -7.7 |
| Flupenthixol | -7.7 |
| Idarubicin | -7.7 |
| Stanozolol | -7.7 |
| Sulindac | -7.7 |
| Cypermethrin | -7.6 |
| Fluspirilene | -7.6 |
| Hydrocortisone | -7.6 |
| Loperamide | -7.6 |
| Mifepristone | -7.6 |
| Moexipril | -7.6 |
| Nelfinavir | -7.6 |
| Penfluridool | -7.6 |
| Pranlukast | -7.6 |
| Acrivastine | -7.5 |
| Cyproheptadine | -7.5 |
| Fexofenadine | -7.5 |
| Barnidipine | -7.4 |
| Clebopride | -7.4 |
| Emetine | -7.4 |
| Haloperidol | -7.4 |
| Loratadine | -7.4 |
| Terfenadine | -7.4 |
| Bromperidol | -7.3 |
| Cisapride | -7.3 |
| Cyclofenil | -7.3 |
| Fulvestrant | -7.3 |
| Indomethacin | -7.3 |
| Quetiapine | -7.3 |
| Cilnidipine | -7.2 |
| Encainide | -7.2 |
| Noscapine | -7.2 |
| Pimethixene | -7.2 |
| Pimozide | -7.2 |
| Pipamperone | -7.2 |

| | |
|---|---|
| Tofisopam | -7.2 |
| Yohimbine | -7.2 |
| Zolantidine | -7.2 |
| Azaperone | -7.1 |
| Butenafine | -7.1 |
| Flecainide | -7.1 |
| Flufenamic-acid | -7.1 |
| Flupirtine | -7.1 |
| ketotifen | -7.1 |
| Opipramol | -7.1 |
| Allylestrenol | -7 |
| AY-9944 | -7 |
| Butorphanol | -7 |
| Carbamazepine | -7 |
| Clozapine | -7 |
| Fluphenazine | -7 |
| Mianserin | -7 |
| Retinol | -7 |
| Apomorphin | -6.9 |
| Chlorotrianisene | -6.9 |
| Phenothrin | -6.9 |
| Tamoxifen | -6.9 |
| Tirofiban | -6.9 |
| Trifluoperazine | -6.9 |
| Chlorprothixene | -6.8 |
| Diazepam | -6.8 |
| Fenspiride | -6.8 |
| Hydroxyzin | -6.8 |
| Mirtazapine | -6.8 |
| Oxyphencyclimine | -6.8 |
| Phenytoin | -6.8 |
| Repaglinide | -6.8 |
| Tibolone | -6.8 |
| Vinpocetine | -6.8 |
| Warfarin | -6.8 |
| Amorolfine | -6.7 |
| Bepridil | -6.7 |
| Dibenzosuberane | -6.7 |

| | |
|---|---|
| Epinastine | -6.7 |
| Fenofibrate | -6.7 |
| Hydroquinine | -6.7 |
| Mebhydroline | -6.7 |
| Quinine | -6.7 |
| Sulpiride | -6.7 |
| Cyclobenzaprine | -6.6 |
| Diphenylpyralin | -6.6 |
| FG7142 | -6.6 |
| Mesoridazine | -6.6 |
| Perphenazine | -6.6 |
| Thioridazin | -6.6 |
| Thioridazine | -6.6 |
| Tiagabine | -6.6 |
| Alaproclate | -6.5 |
| Amitriptyline | -6.5 |
| Benztropine | -6.5 |
| Biperidene | -6.5 |
| Biperiden | -6.5 |
| Clomiphene | -6.5 |
| Desogestrel | -6.5 |
| Hydroxyzine | -6.5 |
| Lynestrenol | -6.5 |
| Maprotiline | -6.5 |
| Meclofenamic_acid | -6.5 |
| Mibefradil | -6.5 |
| Paroxentine | -6.5 |
| Rolipram | -6.5 |
| Vincamine | -6.5 |
| Cloricromen | -6.4 |
| Colchicine | -6.4 |
| Fluoxetine | -6.4 |
| Leukomethylene_blue | -6.4 |
| Mepacrine | -6.4 |
| Naproxen | -6.4 |
| Papaverine | -6.4 |
| Pridinol | -6.4 |
| Sertraline | -6.4 |

| | |
|---|---|
| Triprolidine | -6.4 |
| Aprindine | -6.3 |
| Atropine | -6.3 |
| Betaxolol | -6.3 |
| Diltiazeml | -6.3 |
| Doxepin | -6.3 |
| Ibuprofen | -6.3 |
| Mebeverine | -6.3 |
| Nortriptyline | -6.3 |
| Trihexyphenidyl | -6.3 |
| Amiodarone | -6.2 |
| Antazoline | -6.2 |
| Benzbromarone | -6.2 |
| Citalopram | -6.2 |
| Clemastine | -6.2 |
| Isoxsuprine | -6.2 |
| Lamotrigine | -6.2 |
| Pergolide | -6.2 |
| Perhexiline | -6.2 |
| Phentolamine | -6.2 |
| Procyclidine | -6.2 |
| Carvedilol | -6.1 |
| Disopyramide | -6.1 |
| Lofepramine | -6.1 |
| Norfluoxentine | -6.1 |
| Propafenone | -6.1 |
| Protriptyline | -6.1 |
| Uridine | -6.1 |
| Ambroxol | -6 |
| Dextromethorphan | -6 |
| Fendiline | -6 |
| Harmine | -6 |
| Naphazoline | -6 |
| Tacrine | -6 |
| Tramadol | -6 |
| Triflupromazine | -6 |
| Trimipramine | -6 |
| Venlafaxine | -6 |

| | |
|---|---|
| Amlodipine | -5.9 |
| Cibenzoline | -5.9 |
| Cyclazocine | -5.9 |
| Imipramine | -5.9 |
| Reboxentine | -5.9 |
| Sparteine | -5.9 |
| Bromhexine | -5.8 |
| Clomipramine | -5.8 |
| Diphenhydramine | -5.8 |
| Mitotane | -5.8 |
| Orphenadrine | -5.8 |
| Oxolamine | -5.8 |
| Quinacrine | -5.8 |
| Xylometazoline | -5.8 |
| Benfluorex | -5.7 |
| Brompheniramine | -5.7 |
| Carbetapentane | -5.7 |
| Chloroquine | -5.7 |
| Chlorpheniramine | -5.7 |
| Cloperastine | -5.7 |
| Cyclopentolate | -5.7 |
| Drofenine | -5.7 |
| Fluvoxamine | -5.7 |
| Oxybutynine | -5.7 |
| Oxymetazoline | -5.7 |
| Promazin | -5.7 |
| Promazine | -5.7 |
| Desipramine | -5.6 |
| Ethopropazine | -5.6 |
| Promethazin | -5.6 |
| Promethazine | -5.6 |
| Pyrilamine | -5.6 |
| Tripelennamine | -5.6 |
| Chloropyramine | -5.5 |
| Chlorpromazine | -5.5 |
| Dicyclomine | -5.5 |
| Etomidate | -5.5 |
| Fenfluramine | -5.5 |

| | |
|---|---|
| Proparacaine | -5.5 |
| Ropinirole | -5.5 |
| Alprenolol | -5.4 |
| Bromopride | -5.4 |
| Chlorquinaldol | -5.4 |
| Dienestrol | -5.4 |
| Gabapentin | -5.3 |
| Propranolol | -5.3 |
| Tetracaine | -5.3 |
| LIdocaine | -5.2 |
| Moxisylyte | -5.2 |
| Oxeladin | -5.2 |
| Paraxanthine | -5.2 |
| Camylofin | -5.1 |
| Clonidine | -5.1 |
| Metoclopramide | -5.1 |
| Suloctidil | -5.1 |
| Clenbuterol | -5 |
| Gentisic_acid | -5 |
| Alverine | -4.9 |
| Bupropion | -4.9 |
| D_Mannitol | -4.9 |
| Methapyrilene | -4.8 |
| Phenylmethylsulfonyl_flouride | -4.8 |
| Tulobuterol | -4.8 |
| Memantine | -4.7 |
| Rimantadine | -4.6 |
| L_Leucine_methyl_ester | -4.4 |
| Mecamylamine | -4.3 |
| Amantadine | -4.1 |
| Putrescine | -3.2 |
